# Natural sources and encapsulating materials for probiotics delivery systems: Recent applications and challenges in functional food development

**DOI:** 10.3389/fnut.2022.971784

**Published:** 2022-09-21

**Authors:** Shubhi Singh, Rishibha Gupta, Sonam Chawla, Pammi Gauba, Manisha Singh, Raj Kumar Tiwari, Shuchi Upadhyay, Shalini Sharma, Silpi Chanda, Smriti Gaur

**Affiliations:** ^1^Department of Biotechnology, Jaypee Institute of Information Technology, Noida, India; ^2^School of Health Sciences, Pharmaceutical Sciences, The University of Petroleum & Energy Studies (UPES), Dehradun, India; ^3^Department of Allied Health Sciences, School of Health Sciences and Technology, The University of Petroleum & Energy Studies (UPES), Dehradun, India; ^4^Sunder Deep Pharmacy College, Ghaziabad, India; ^5^Department of Pharmacognosy, Parmarth College of Pharmacy, Hapur, India

**Keywords:** probiotics, encapsulation, nutraceutical, functional food, viability

## Abstract

Probiotics are known as the live microorganisms which upon adequate administration elicit a health beneficial response inside the host by decreasing the luminal pH, eliminating the pathogenic bacteria in the gut as well as producing short chain fatty acids (SCFA). With advancements in research; probiotics have been explored as potential ingredients in foods. However, their use and applications in food industry have been limited due to restrictions of maintaining the viability of probiotic cells and targeting the successful delivery to gut. Encapsulation techniques have significant influence on increasing the viability rates of probiotic cells with the successful delivery of cells to the target site. Moreover, encapsulating techniques also prevent the live cells from harsh physiological conditions of gut. This review discusses several encapsulating techniques as well as materials derived from natural sources and nutraceutical compounds. In addition to this, this paper also comprehensively discusses the factors affecting the probiotics viability and evaluation of successful release and survival of probiotics under simulated gastric, intestinal conditions as well as bile, acid tolerant conditions. Lastly applications and challenges of using encapsulated bacteria in food industry for the development of novel functional foods have also been discussed in detail too. Future studies must include investigating the use of encapsulated bacterial formulations in *in-vivo* models for effective health beneficial properties as well as exploring the mechanisms behind the successful release of these formulations in gut, hence helping us to understand the encapsulation of probiotic cells in a meticulous manner.

## Introduction

World Health Organization (WHO) and the Food and Agriculture Organization (FAO) have defined probiotics as the live microorganisms, including bacteria and yeasts, which upon consumption in adequate amounts confer health promoting effects to the host. *Lactobacillus* and *Bifidobacteria* are most commonly used probiotics which are either naturally present in food or added to increase the nutritional as well as functional quality of food (1). The consumption of probiotics have been associated with several therapeutic effects like protection against diarrhoeal diseases, inflammatory disorders, hypercholesterolemia as well as exhibit anticancer, anti-diabetic and anti-oxidative effects by enhancing the host immunity, strengthening the gut barrier and production of immune protective markers (2). Probiotics exists in liquid, paste, powdered form and are available in sachets as well as capsules. These have been widely used up in the food industry in development of novel functional foods, medicinal foods as well as dietary supplements. WHO and FAO suggests that in order to provide satisfactory health benefits to host, the viability of the probiotic cells should be more than 10^6^ log CFU/ml or g in food (3). An emerging solution to compromising viability of ingested probiotics is encapsulation, and is endorsed by research fraternity as well as food industries. However, encapsulation is also not without drawbacks. Probiotic vulnerability to encapsulation parameters and the gut harsh conditions on ingestion—pH, temperature and oxygen tension, are still a serious concern. (4). To overcome these existing problems, several approaches have been proposed to increase the viability rates by decreasing the harmful environmental stresses. For instance; selection of acid and alkaline probiotic strains, using oxygen impermeable vessels, addition of micro and macro nutrients as well as encapsulating the probiotics, are some of the proposed approaches to increase the viability. Amongst these, encapsulation techniques have gathered great attention as this involves the entrapment of material in a matrix of polymeric membrane without affecting its biological activity. In this technique, the active materials are entrapped in the polymeric capsules which further prevent its deterioration from harmful environmental effects, leading to improved viability rates as well as successful release to the target sites. This technique has successfully been utilized in food industry, pharmaceutical industry, textile industry, cosmetics etc., (5). This paper extensively discusses the various different types of techniques involved in encapsulation of probiotics as well as the materials required for encapsulation. Moreover, the factors affecting the viability rates of probiotics during encapsulation have also been discussed in detail. Direct application of encapsulated probiotics in food industry, especially in development of novel functional foods with enhanced health beneficial properties are also comprehensively discussed in this paper.

## Encapsulating techniques: Properties, advantages, and disadvantages

**Figure 1:**
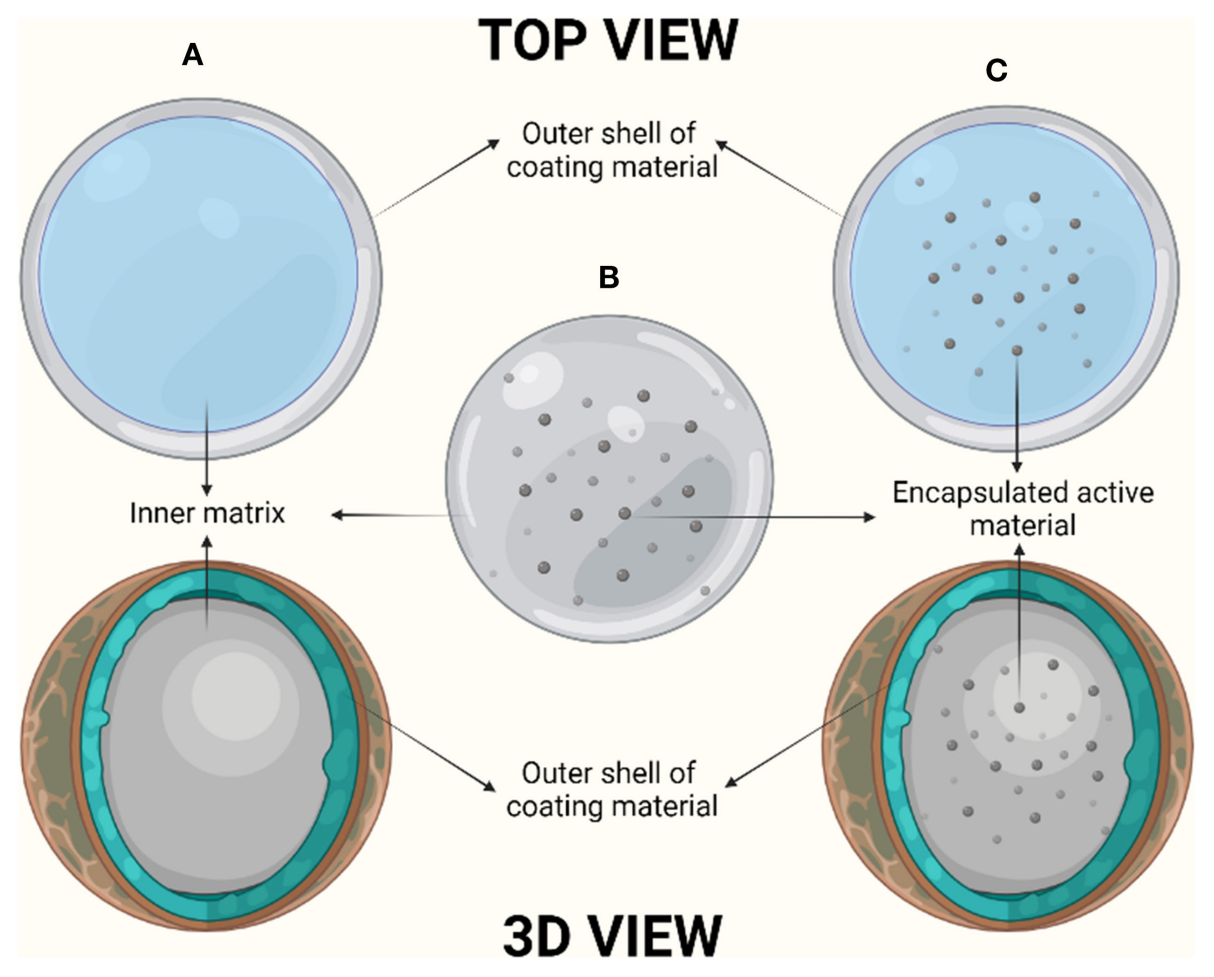
Diagrammatic representation of encapsulation systems: **(A)** Reservoir, **(B)** Matrix, and **(C)** Coated matrix.

Encapsulation is defined as the process of entrapment of one material by another material. The material which is being entrapped is known as active material, filling material and internal material whereas the material which is being used to entrap the former one is called carrier, coating membrane, outer membrane and matrix. Based on the type of encapsulation method, there are majorly three variants of encapsulates: reservoir (microcapsule), matrix (microparticle) as well as coated matrix (multi- wall structure) (6) (Figure 1). Reservoir is defined as the encapsulation method where the active material is being capsuled by the outer layer of carrier material. The other encapsulation method, matrix, has the active material dispersed evenly over and on the surface of the carrier material. The coated matrix is defined as the method of encapsulation, where the capsuled active material is coated from additional coating material (7).

### Emulsification

**Figure 2:**
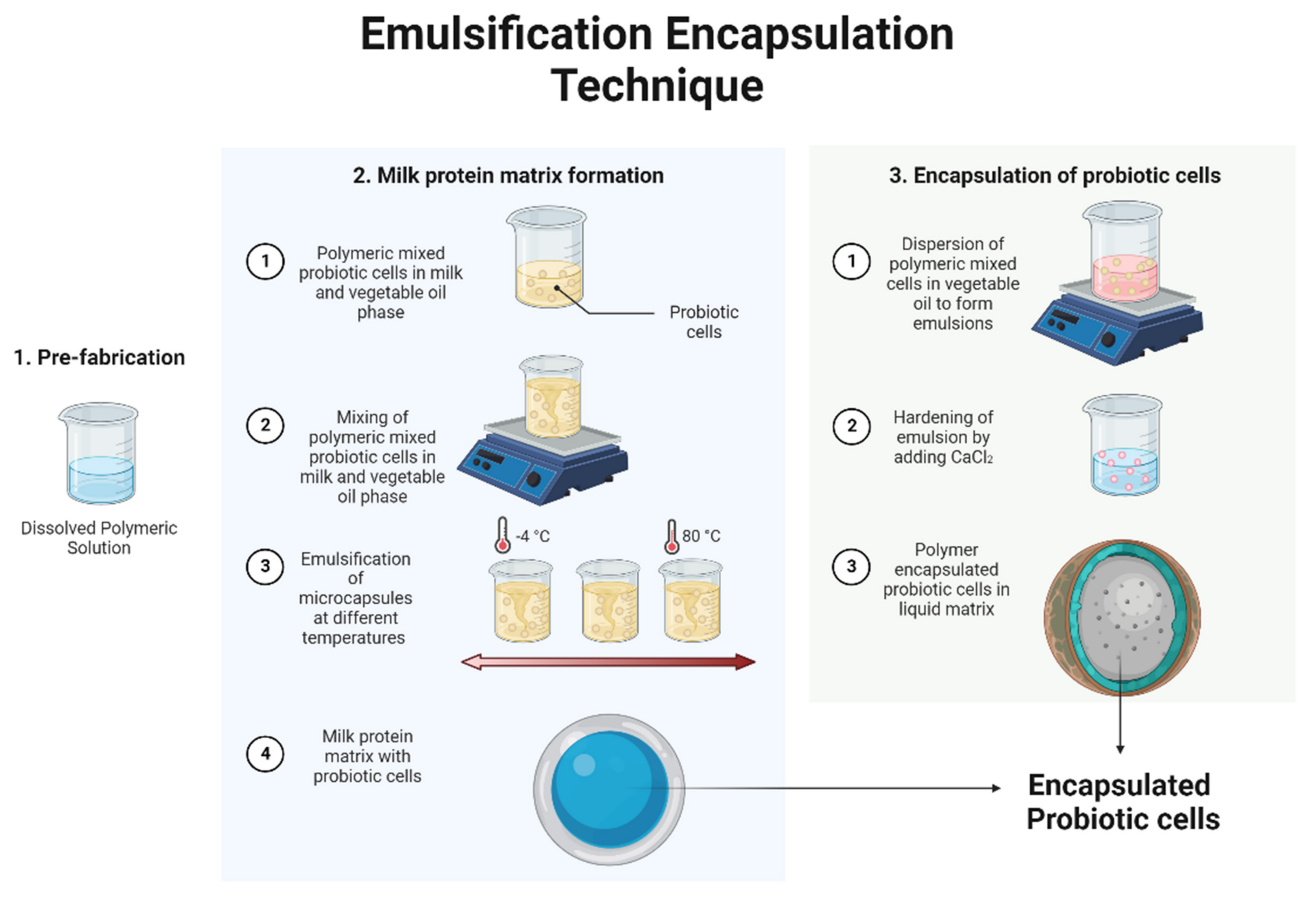
Diagrammatic representation of emulsification encapsulation technique.

Emulsification is a chemical technique to encapsulate active materials using the two phase systems. The discontinuous phase contains cell polymer hydrocolloid suspension of microorganisms whereas the continuous phase contains oil solution to form the emulsions. The discontinuous phase containing the active material is added to larger volume of continuous phase with regular stirring. The mixture of two phases forms the emulsion which is hardened by addition of CaCl_2_. Once the emulsion is formed, the outer layer must be removed to make tiny particles of encapsulated active material (8). This chemical method of encapsulation has been applied in encapsulating the probiotic cells (Figure 2). The main advantage of this technique is higher survival rates of probiotic bacteria as well as ease of scale-up at industrial levels (9). Moreover, this technique helps in production of encapsulated bacterial capsules with targeted size by optimizing the agitation speed and varying the water:oil ratio to produce the emulsions. Generally, the size of the microcapsules varies from 0.2 μm −8 mm (10). Encapsulating probiotic bacteria using emulsification is a well-established and widely used technique in food industry and has been discussed in later sections. One disadvantage with this technique is the application of additional polymeric layer to protect the encapsulated material to provide added protection. It is known that the classical way of encapsulating probiotics using emulsification makes use of alginate, carrageenan, as well as xanthan gum as outer coverings; which are not acceptable in food industries (11). Hence the solution is to use the milk based proteins as outer coating materials. Additional advantage of using the milk proteins as coating materials is that they have good gelation properties as well as act as natural carriers for probiotics. For gelation purposes, the proteolytic enzyme complex- rennet has been applied which successfully produces the casein micelles by splitting apart k- casein (12). These micelles are known to form gels at temperature above 18°C, hence protecting the encapsulated probiotic cells from outside environment (13). Besides this, using the emulsified encapsulated probiotic cells for fermentation industry with better productivity interfacial polymerization technique is used. In this technique, once the emulsions are formed, the biocompatible agent, like alginate, chitosan, carrageenan and gelatin are added to the continuous phase which encapsulates the bacteria from the discontinuous phase by forming thin layer droplets (14).

### Spray drying

**Figure 3:**
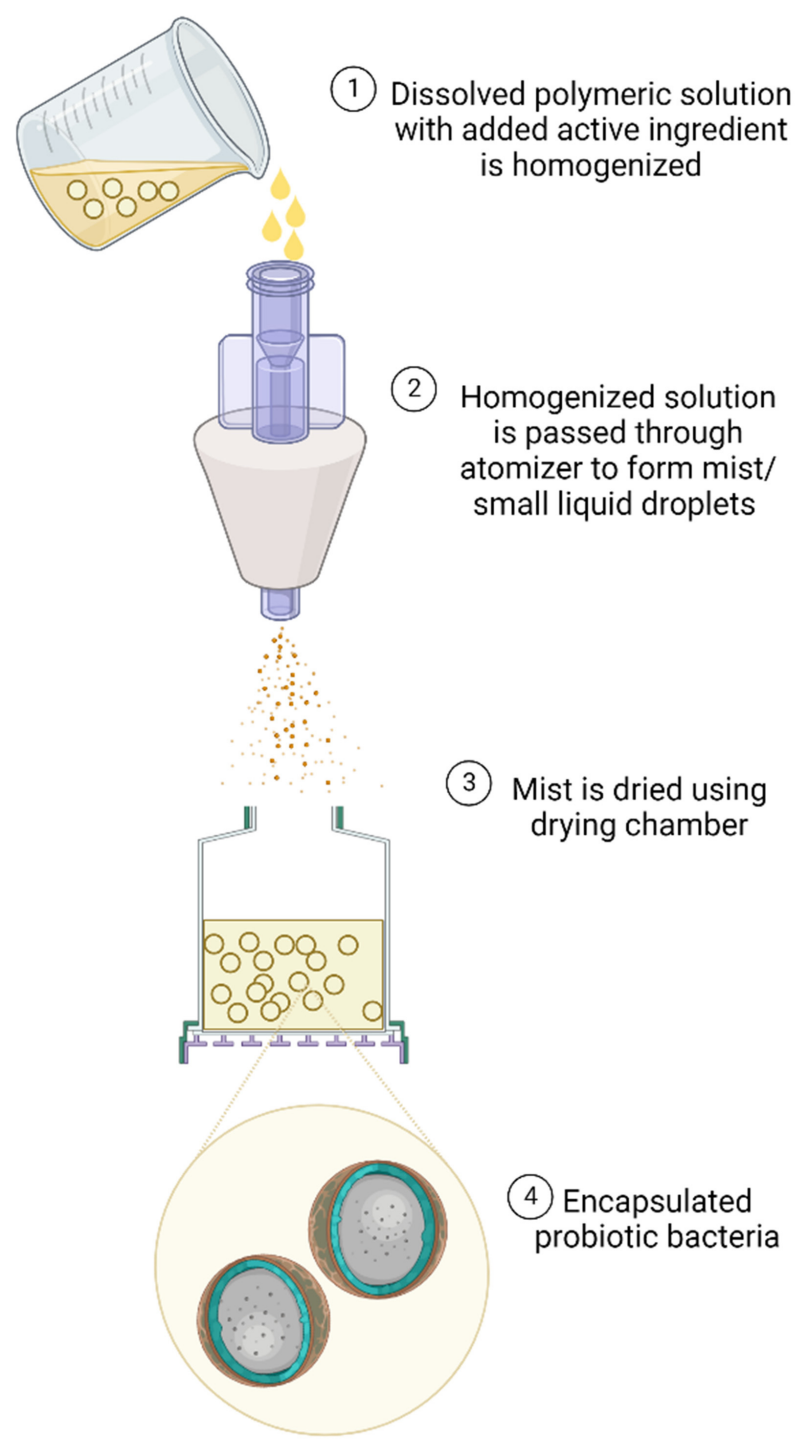
Diagrammatic representation of spray drying encapsulation technique.

Spray drying is another dispersion technique to encapsulate active materials using two phase systems—liquid and air. In this technique, the solution of active material and the dissolved polymer is prepared and homogenized. The prepared solution is atomized using the atomizer to form mist/small liquid droplets which are then dried using hot air or nitrogen gas in the drying chamber at the temperature 150- 250° C (15). This procedure helps in evaporation of the bonded solvent followed by transferring the dried encapsulated material to the cyclone separator for further recovery. Generally, the polymeric solution used in this technique contains maltodextrin, granulated starchy solution, alginate, guar gum, xanthan gum as coating materials to encapsulate the active materials (16). Spray drying method is economic method with minimum energy utilization as compared to any other methods of encapsulation, producing the microcapsules of size 10–150 μm (17). This method has been widely used to encapsulate the probiotic bacteria too (Figure 3). This technique needs proper control and adjustments of inlet and outlet temperatures to maintain the viability of the probiotic cells. The only disadvantage with technique is that it has limited compatibility with only selected strains of probiotics which are tolerant to high range of temperature during the drying step (18). It is known that during the drying process, there is the reduction in the water activity and hence the bacteria become resistant to osmotic stress. The encapsulated bacteria are known to maintain their viability by accumulating the layer of carbohydrates as well as other amines. To provide protection to encapsulated bacteria from higher temperatures, the dryers are often equipped with cooling devices to lower the temperature of prepared microcapsules (19).

### Lyophilisation (freeze drying)

This is the most commonly used technique for encapsulation of probiotics. This method involves higher freezing rates to avoid the damage to probiotic cells from small ice crystals. Freeze drying involves three steps of freezing, primary drying and secondary drying. In first stage, at lower freezing temperatures, the chemical and osmotic changes occur due to crystallization of water. The frozen water crystals are removed in primary drying due to sublimation process under vacuum conditions. The residual unfrozen water is again removed by desorption during secondary drying (20). Though this method is very convenient to carry out and provides microcapsules with larger surface area as compared to spray dried capsules, but the formation of ice crystals often damages probiotic cells' membranes cellular and surface proteins as well as reduces the water content of the cell, compromising the cellular viability (21). Moreover, this method requires more energy consumption and longer processing time (22). To overcome the demerits of this technique, some of the cryo- protectants have been added to during the process of freeze drying. The carbohydrates rich compounds like lactose, polyethylene glycol and sucrose have been extensively used as cryo-protectants which increase the unfrozen water and provide more space to probiotic cells to survive. These are also known to reduce the cellular damage caused by physical and osmotic stress (23). Another disadvantage of freeze drying is the necessity to select probiotic bacteria tolerant for low process temperatures. Researchers recommend a pre-stress conditioning phase, exposing bacteria to low temperatures to acclimate them as well as optimizing the process conditions to ensure viability of the cells (24).

### Extrusion

**Figure 4:**
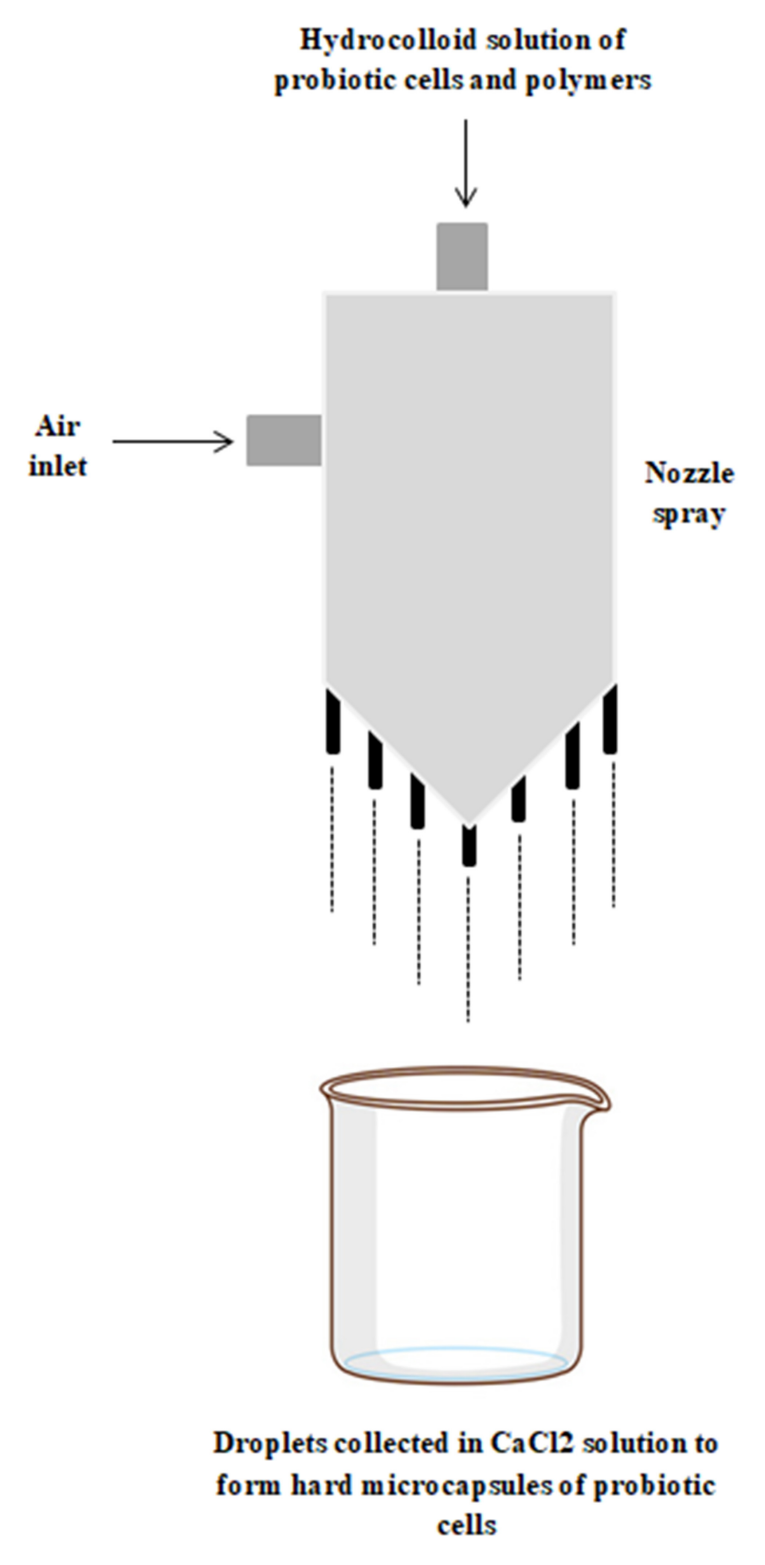
Diagrammatic representation of extrusion encapsulation technique.

Extrusion is another dispersion technique that is low cost and widely used. It involves mixing of probiotic cells into the polymeric hydrocolloid solution. The mix is passed through the nozzle of spray machine and the small droplets of the mix are projected into a solution of CaCl_2_ which results in gelation by cross linking mechanism. Though this technique is easy to scale up at industrial levels, there are several demerits of this technique (25). The number of process variables—temperature, concentration, flow rate and viscosity of the polymer and probiotic mix, diameter of the nozzle and drop height, to be controlled is high and affects the size of microcapsules formed (Figure 4). To overcome these disadvantages, advanced extrusion technique—prilling was introduced. In this technique, the droplets are formed under the controlled environmental conditions by applying vibrations to the nozzle sprayer. Prilling provides the control on the size of the beads by fluctuating the applied electric potential. This also reduces the cost by minimizing the dependency on additional organic solvents. Another advanced extrusion technique is centrifugal extrusion which involves the application of concentric orifices at the outlet of the nozzle sprayer and hence producing the microcapsules of desired size (26).

### Electrospraying

Electrospraying offsets the disadvantages of using solvents and higher temperatures in previously mentioned techniques. In this technique, high speed and voltage is applied to form the microcapsules. This reduces the chances of toxicity of encapsulated material caused due to usage of solvents. The electric field is applied on the polymeric solution containing the probiotics to form capsules of size ranging from nano to micrometer, without the requirement of higher temperatures (27). There are several advantages associated with this technique like high versatility, simplicity and easy scale up. Since, there is no involvement of heat; there is minimum thermal damage to encapsulated probiotic cells in this technique (Table 1) (36).

**Table 1:** Advantages and disadvantages of encapsulation techniques.

**Encapsulation technique**	**Capsule size**	**Advantages**	**Disadvantages**	**References**
Emulsification	0.2 um−8 mm	• Higher survival rates of bacteria • Control release of capsules with desired size	• Variation in shape of capsules • Addition of extra polymer layer to protect encapsulated cells	(9) (28)
Spray drying	10–120 um	• Easy scale- up with mass production • Inexpensive • Economic process with minimum energy utilization	• Higher temperatures results in loss of viability of cells • Incorporation of additional cooling devices	(29) (30)
Lyophilisation	-	• Provides larger surface areas of microcapsules • Most widely used method for sensitive materials	• Formation of ice crystals damage probiotic cells • Need of additional cryo- protectants	(31) (32)
Extrusion	1,000–5,000 um	• Easy scale- up with mass production • Inexpensive • Continuous process	• Large size capsules formation • Complex setting of physico- chemical parameters	(33) (34)
Electrospraying	5 um- 1,000 um	• High voltage and speed reduces chances of toxicity due to solvents • Inexpensive • Control release of capsules with desired size	• Shear forces affect the viability of encapsulated probiotics	(35)

## Potential encapsulating materials in food system

The choice for encapsulation material is a challenging task. Apart from economic considerations the material should be edible and chosen such that it ensures the viability of probiotics at all stages of its development: from processing to storage to its targeted delivery in the GI tract. It has been commonly observed that a matrix showing gel like properties with high content of dry matter are generally more suitable (37). Nature of encapsulating material has a crucial role in determining encapsulation efficiency. A number of polymeric materials including polysaccharide hydrocolloids (alginate, xanthum, chitosan, carrageenan, cellulose), fats and proteins have been used for microencapsulation (24). Alginate is one of the most widely used materials due to its characteristics property to resist pH 2–2.5 (gastric pH) and expand at neutral to alkaline pH (38) (Table 2). However, the application of alginate is limited by high porosity. Therefore, use of other co-encapsulating materials has been proposed in a number of studies. A study by Iqbal and co-workers shows the potential use of chitosan or sodium alginate along with whey protein to form a double coating improving the viability of *Bifidobacterium bifidum ATCC 35914* in simulated gastric environment from <1.87 log units of free cells after 1.5 h to more than 10^6^Log CFU/mL after 2 h incubation (pH 2) (41). Polysaccharides have been classified into five distinct categories (46) on the basis of their structural role in microcapsulation as:

1. Polysaccharides that can be induced to form gels in presence of ions such as alginate, pectin in presence of Ca^2+^ ions or carrageenan via K^+^ ions
2. Polysaccharides that reinforce the structure and are resistant to acid and enzyme actions such as gellan gum
3. Polysaccharides that are selectively dissolute only in enteric environment such as cellulose acetate propionate
4. Polysaccharides with charges that can interact with opposite charges such as chitosan combining with alginate or pectin
5. Polysaccharides with prebiotic potential.

**Table 2:** Examples of various encapsulating materials and its applications in food systems.

**Encapsulation material**	**Prebiotic**	**Probiotic**	**Encapsulating system**	**Food matrix**	**Reference**
Whey protein isolates + Dextran conjugates prepared by Maillard-based glycation	-	*Lactobacillus plantarum*	High molecular weight conjugate	Kefir	(39)
1.5% Alginate+ Persian gum (0.5%)	2% Inulin	*L. lactis ABRIINW-N19*	Microencapsulated bead	Orange juice	(40)
6% Sodium Alginate/ 0.8% Chitosan +Whey protein concentrate (5%)	-	*Bifidobacterium bifidum* ATCC 35914	Double coated microbeads	-	(41)
Tarkhineh formulations	-	*Lactococcus lactis* KUMS-T18	Probiotic drops	Potato chips	(42)
Cocoa powder +Sodium Alginate (10:1) and Cocoa powder +Na Alginate + Fructooligosaccharides (10:1:2)	FOS	*Lactobacillus acidophilus* (La5), *L. rhamnosus* (LGG), *L. sanfranciscensis, L. plantarum, L. casei* 431, *Bifidobacterium animalis* subspp. Lactis (Bb12), and *Streptococcus thermophilus*.	Emulsion based beads using cocoa powder as an admixture	Chocolate	(43)
Xanthan gum (2%), Maltodextrin (1%), Sucrose (0.5%), Sunflower oil (0.1% v/v) Tween 80	-	*Lactobacillus acidophilus, Lactobacillus rhamnosus* and *Bifidobacterium longum* in 1:1:1 ratio	Microencapsulated powder was used in cream which was then applied between the biscuits	Cream biscuit	(44)
Sodium Alginate (2%) and Arabic gum (5%)	-	*Lactobacillus plantarum*	-	Rose Petal Jam	(45)

It should also be taken into consideration that food is a complex matrix where presence of many components such as antimicrobial compounds, additives, polyphenols, prebiotics and many more can have a direct effect on probiotics survivability especially during storage (4). A recent study shows that the viability of *Lactobaccilus plantarum* decreased in rose petal jam due to inherent low pH and water activity of jam. However, microencapsulation with 3.5% or 5% Arabic gum and 2% sodium alginate improved the storage stability of these probiotics and were detected at acceptable levels even after 90 days of storage at both room and refrigerated temperatures (45).

Prebiotics is defined as “*a substrate that is selectively utilized by host microorganisms conferring a health benefit*” (4). Some of the commonly used prebiotics are fructooligisaccharides (FOS), inulin, fructans, galacto-oligosaccharide. However, newer prebiotic sources are also being constantly explored such as dietary fibers, carbohydrates with different glycosidic linkages or monosaccharide content that can be used with or without modifications (4), exopolysaccharides such as mucilages, natural bioactive polysaccharides such as lentinan, psyllium and many more (46). Extracts of natural plants are also been increasingly explored for co-encapsulation in microencapsulating system such as onion extract (47), blueberry extract (48) for enhanced functional properties. Nami and co-workers studied the effect of incorporation of two prebiotics FOS and inulin in an alginate-persian gum-based hydrogel system for probiotic incorporation of *Lactococcus lacti* ABRIINW- N19 in orange juice. The study indicates that prebiotics got integrated in the polymeric two layered structure formed by the hydrogel. Both the prebiotics had good encapsulation efficiency, GI survivability and storage stability with decreased malic acid formation and sugar consumption and increased buffering capacity in comparison to free cells in orange juice. The viability decrease in encapsulated cells was only 1.46 log CFU/g compared to 6.52 log CGU/g for free cells after 2 h of simulated GI conditions (pH 2.5). However, in terms of release the dense structure of inulin used at higher percentages resulted in slow release that is observed completely only after 2 h (40). In another study, FOS isolated from banana peel was used along with sodium alginate showed an improvement in encapsulation efficiency of *Lactobacillus rhamnosus*. Besides, the incorporation of FOS in sodium alginate at 50 percent significantly enhanced viability with log reduction of 1.4 log CFU/mL as compared to 7.5 log CFU/mL for free cells (49).

In a recent interesting study, use of black waxy rice was explored as a prebiotic source in place of rice starch with an aim to utilize its' resistant starch which has slow digestibility and additionally has bioactive anthocyanins. These prebiotics have previously been reported to enhance growth of good bacteria particularly *Bifidobaterium* and *Lactobacillus* species and also impart favorable sensorial characteristics during *Lactobacillus* based fermentation. Therefore, the enzyme and heat moisture treated black waxy rice was used to encapsulate *Lactobacillus plantarum* and then incorporated in yogurt. Increased short chain fatty acid production and favorable growth of other probiotic species in yogurt suggests that resistant starch of rice could be further explored as prebiotic in synbiotic yogurt development (50).

In recent years, focus has also been on developing encapsulation material providing improved targeted delivery. The ability of certain materials to adhere to mucosal membranes can provide enhanced retention of probiotics in GI tract and thus can have direct impact on the bioavailabilty, administration frequency and targeted action of probiotics (51). An interesting study by Phuong and co-workers explored the potential of different materials including chitosan, chitosan coated alginate, alginate and alginate-resistant starch on gastric adhesion of probiotic bacteria *Lactobacillus plantarum*. The chitosan coated alginate showed mucoadhesive properties similar to chitosan and thus can be used as a system for targeted delivery of probiotic to gastric epithelium (52).

## Factors affecting probiotics viability during encapsulation

Evidence suggests nearly 10^6^ fold decline in the colony forming units in several commercial probiotic formulations within the first 5 min of exposure to simulated gastro-intestinal tract (GIT) conditions (53). Vulnerability to the harsh conditions during food processing/formulation, storage and in the GIT are key factors compromising the viability of beneficial probiotic rendering them ineffective. Further, emphasis is also placed on the three dimensional characteristics of the encapsulated particle in influencing the viable cell count. Herein, we discuss the various factors compromising the viable cell count:

### Heat

Largest fraction of commercial strains of probiotics known today have established thermal susceptibility, as the optimal temperature for probiotic viability evolved to be the human body temperature. Mechanistic introspection of this declined viability due to low versus high temperatures exposure manifests via exacerbated membrane porosity leading to leakage of intra-cellular contents, and thermal stress mediated inactivation of critical molecular machinery (polymerases), respectively (54–56).

Food processing operations constituting probiotics are varied and expose the constituting microorganisms to widely different temperatures. At one end of the temperature spectrum are procedures such as freeze drying where cells are exposed to extremely low temperatures (up to −40°C), whereas spray drying, sterilization and pasteurization involve exposure to temperatures more than 60°C (57, 58). Diminished decimal reduction time and a 4–6 log reduction is reported in fruit juice inoculated with various species of *Lactobacillus* and *Bifidobacterium* on a 30 s exposure to 76°C (59). In fact, compromised viability is observed despite the encapsulation - *Lactobacillus reuteri* capsules dried at 55 °C led to decrease in viability from 1.6 × 10^9^ CFU g ^−1^ to 2.5 × 10^7^ CFU g^−1^ (60). Composition of the encapsulating matrix is a key determinant of the thermal stress and cell death post-encapsulation. *Bifidobacterium pseudocatenulatum* CECT 7765 is an emerging probiotic bacterium with demonstrated health benefits in alleviating inflammation, vascular and neuroendocrine distress, metabolic syndrome incidence etc, but it is particularly heat sensitive (61, 62). Alehosseini and co-workers prepared agarose-based freeze-dried capsules via an “oil induced biphasic hydrogel formation” technique (agarose in combination with alginate, whey protein concentrate, gelatin) of *Bifidobacterium pseudocatenulatum*. Post-encapsulation, freeze drying conferred a 40–75% enhanced survival of the *Bifidobacterium pseudocatenulatum* CECT 7765 as compared to directly freeze-dried probiotic solution. Especially the combination of agarose with whey protein led to a marginal 1 log unit decline in cell viability over 2 months (63). Whey protein, milk proteins and gelatin have been demonstrated to confer protection to probiotic bacteria against thermal stress as well as other adverse ambient conditions in lieu of their amino acid composition which limits heat transfer at high temperatures and ability to form a viscous cell coating, protecting against ice crystal puncturing at low temperatures such as in lyophilization or freeze drying (64–66). Inclusion of sugars like lactose and trehalose has also been demonstrated to have protective abilities against thermal stress due to their ability to form stabilizing hydrogen bonding with the structural proteins constituting the plasma membrane and maintain its integrity despite displacement of water molecules due to thermal stress, during spray drying and storage (67, 68). Inclusion of low-melting-temperature fats into the encapsulating matrix limits heat transfer to the probiotic cells in the shell, as the fats melts and absorbs the heat, limiting the high temperature shock (69).

An alternate emerging strategy to counter thermal inactivation of probiotics during encapsulation is to expose the probiotic culture to an acclimatizing heat treatment at 50–52 °C for approximately 15 min to trigger the heat shock proteins and eventually confer protection against near-lethal temperature during food processing operations. The method has shown promising results in various heat sensitive strains of *Lactobacillus* spp (70).

A novel potential category of probiotic bacteria is soil-based organisms, *Bacillus* spp. being a prime example. The bacteria and its spores are heat resistant, and confer several health advantages (reviewed elsewhere by Lee and co-workers) (71). Survivability of *Bacillus coagulans* post-encapsulation is higher than the more common *Lactobacillus spp*. during spray drying as well as freeze drying. Nonetheless the material of the encapsulating matrix is the determinant in survivability during the processing procedures, skimmed milk conferring maximum protection and xanthan gum the least (72).

### Oxygen toxicity

Differential oxygen tolerance of probiotic bacteria was reported as far back as in 1969 in different strains of *Bifidobacteria* (73). The microaerophilic or anaerobic nature of gut bacteria is attributed to the absence of electron transport chain, the ultimate cellular electron sump, leading to partial reduction of oxygen to form the cytotoxic hydrogen peroxide. This is more popularly known as oxygen toxicity (74). High levels of oxygen incorporated in food products during processing and preparation is an accepted factor compromising the viability of probiotic formulations. However, the physical isolation of microbial cells during encapsulation shields the cells from direct exposure to high oxygen levels permeating the formulation. Enhanced oxygen tolerance in encapsulated probiotics has been demonstrated in several investigations, notably, in the dairy industry for products such as yogurt, ice-cream, cheese etc (24, 75). Alginate encapsulated *L. acidophilus* and *Bifidobacterium* spp. are reported to have anoxic regions at the center of the encapsulated particle, creating a favorable microenvironment for survival of the cells. Significantly higher viable cell counts were observed in encapsulated cells vs. free cells, as a proof of concept that encapsulation shields from toxicity (76, 77).

An alternative strategy to enhance the oxygen tolerance of encapsulated cells can be to co-encapsulate an oxygen-consuming bacterial strain such as *Streptococcus thermophilus*, and create a favorable microenvironment for microaerophilic/anaerobic bacterial survival (24, 78). Additional precautions can be taken by setting up anaerobic environment during the encapsulation process, deoxygenating the encapsulating matrix in liquid phase (79) and adding anti-oxidants such as L-cysteine to sustain the microaerophilic and anaerobic organisms (77).

### pH

The optimal pH for the viability and growth of probiotic bacteria is pH 6–7, the pH in the human colon microenvironment. The gastric pH, compromises probiotic survival by raising the cytosolic concentration of H^+^ ions which further interfere with activity of ATPase as well as denaturing the structural proteins and enzymes (80).

Alginate is a much-favored encapsulating matrix for probiotic formulations due to its pH responsive nature (81). Interestingly, a synbiotic formulation of a multi-particulate system comprising of poly (d,l-lactic-co-glycolic acid) (PLGA) microcapsules containing galacto-oligosaccharide in an alginate-chitosan matrix with *Bifidobacteria* enhanced the viable cell counts several log higher than a simple alginate-chitosan microencapsulation system (82). Recently, Pupa and co-workers demonstrated the efficacy of three double-microencapsulating formulations in preserving the probiotic viability of various *Lactobacillus plantarum* strains, *Pediococcus pentosaceus* 77F, and *P. acidilactici* 72N in the face of acid stress, bile stress and anti-bacterial activity. Namely, the double encapsulation comprised of alginate (1.5%) and chitosan (0.5%) and the microcapsules were formed *via* extrusion, emulsion, and spray drying. Spray-dried double microencapsulated formulation emerged as the most efficacious in preserving probiotic viability even after 6 months of storage at room temperature (83).

### Bile stress

Besides the low pH, the GIT enzymes—lipases, proteolytic enzymes and amylases, in combination with the bile salts in the small intestine contribute to the harsh environment compromising the viability of cells in probiotic microencapsulated formulations (84). Bile acids in the small intestine are anti-bacterial and have detergent action which disrupts the bacterial cell membranes and membrane localized proteins, besides damaging the cellular nucleic acids (85). Differential bile tolerance amongst different probiotic bacteria relies on the expression of bile salt hydrolase enzyme (BSH). BSH positive bacteria are more tolerant to bile exposure. Besides BSH, bile salt efflux system also exist in various probiotic bacteria as a mechanism to cope with bile stress during passage *via* the small intestine (54, 86, 87). An interesting correlation between bile tolerance and antibiotic susceptibility of lactobacilli has also been reported, bile exposure renders lactobacilli susceptible to clinical regimes of antibiotics (88). Thus, shielding the probiotic bacteria from bile exposure is a favorable approach.

Khosravi Zanjani and coworkers formulated *Lactobacillus casei* and *Bifidobacterium bifidum* into alginate-gelatinize starch microcapsules with chitosan coating and observed significant protection from the simulated intestinal juice composed of pancreatin and 4.5% bile salts at pH 8.0 (89). Spray dried casein based emulsion of *Bifidobacterium* spp. and *Lactobacillus acidophilus* and several other commercial probiotic supplements was reported to have higher bile tolerance than non-encapsulated bacteria (90). Singh and co-workers formulated exo-polysacchride, alginate microcapsules containing *L. acidophillus* and other probiotic strains, and demonstrated enhanced viable cell counts in the simulated intestinal conditions (91).

## Applications in functional food development and challenges

Probiotics have been considered as an important addition to food since ancient times. The selection of the probiotic strains often depends on the health beneficial properties as well as characteristics of the final product. Several strains of probiotics have been added traditionally to dairy products like cheese, buttermilk as well as dairy drink to carry out the fermentation process. Apart from the dairy products, probiotics have also been added to non- dairy food products like meat, cereals, vegetables and fruit juices as well as bakery items (92). As discussed in previous sections, the process of encapsulation of probiotics in food is a crucial decisive step to ensure viability and functionality of probiotics in functional foods and nutraceuticals. Herein we discuss examples of commercialized food products where encapsulated probiotics have been incorporated successfully: (Table 3).

**Table 3:** Applications of encapsulated probiotics in functional food development.

**Food product**	**Encapsulation technique**	**Encapsulating materials**	**Probiotic strain**	**References**
Yogurt	Extrusion	Sodium Alginate, Carrageenan	*L. acidophilus*	(93)
Yogurt	Emulsification	Xanthan, Chitosan	*Bifidobacterium BB01*	(94)
Yogurt	Emulsification	Alginate, Calcium Chloride	*Lacticaseibacillus rhamnosus GG*	(95)
Yogurt	Emulsification	Sodium Caseinate, Gellan Gum	*Lactobacillus paracasei*	(96)
Yogurt	Spray drying	Gum Arabic, Synsepalum dulcificum	*Lactococcus lactis Gh1*	(97)
Yogurt	Spray drying	Alginate, Xanthan	*Bifidobacterium-BB12*	(98)
Cheese	Emulsification	β-glucan, Phytosterol	*Lactobacillus rhamnosus*	(66)
Cheese	Emulsification	k- Carrageenan, Sodium Alginate	*Bifidobacterium bifidum*	(99)
Cheese	Extrusion	Wheat Starch, Camel Milk Protein	*Pediococcus pentosaceus*	(100)
Cheese	Emulsification	Skim Milk Powder, Rennet, Transglutaminase, Sodium Caseinate	*Lactobacillus paracasei*	(96)
Cheese	Spray drying	-	*Lactiplantibacillus plantarum 564, Lactiplantibacillus plantarum 299v*	(101)
Bread	Emulsification	Sodium Alginate, Fish gelatin	*Lactobacillus acidophilus LA-5*	(102)
Cupcake	Emulsification	Sodium Alginate, Maltodextrin, Pectin	*Lactobacillus plantarum ATCC8014*	(103)
Wheat Buns	-	Sodium Caseinate, Chia Mucilage	*Limosilactobacillus fermentum NKN51, Lactobacillus brevis NKN52*	(104)
Gluten free Bread	Spray drying	Tragacanth gum, Sago starch	*Lactobacillus acidophilus, Lactobacillus plantarum*	(105)
Bread	Encapsulation	Reconstituted Skim Milk, Gum Arabic, Maltodextrin, Inulin	*Lactobacillus plantarum*	(106)
Juice powder	Spray drying	Maltodextrin	*Lactobacillus rhamnosus, Lactobacillus casei, Lactobacillus plantarum*	(107)
Maoluang Juice	Spray drying	Inulin and *Tiliacora triandra* gum	*Lactobacillus casei 01, Lactobacillus acidophilus LA5*	(108)
Litchi Juice	Spray drying	Maltodextrin, Fructooligosaccharide, Pectin	*Lactobacillus plantarum*	(109)
Juice	Spray drying	Maltodextrin, Inulin	*Bifidobacterium animalis ssp. lactis BB-12*	(110)
Grape Juice	Emulsification	Alginate	*Lactobacillus acidophilus, Bifidobacterium bifidum*	(111)

### Yogurt

Yogurt is one of the first dairy products used to incorporate probiotic bacteria. Several studies have been reported in literature making use of encapsulated probiotic cells without affecting the traditional way of yogurt preparation. However, the survivability of incorporated bacteria is often affected by high acidic environmental conditions. Hence, the better alternate is to use micro capsulated probiotic cells. For instance, L. *acidophilus* was incorporated in the yogurt during its preparation. The addition of free probiotic showed that the bacterial survival decreased from 9.97 log cfu/ml on first day to 6.12 log cfu/ml on day 28. To increase the viability of probiotics, the bacteria were encapsulated using sodium alginate and carrageenan using the extrusion technique. Encapsulated bacteria were released into the yogurt and it was observed that encapsulation using sodium alginate as well as carrageenan decreased the viability of encapsulated bacteria from 9.91 log cfu/ml to 8.74 log cfu/ml and 9.89 log cfu/ml to 8.39 log cfu/ml respectively from first day to day 28. The results showed that in both the cases the viability of probiotic bacteria was decreasing. However, the decrease in viability of bacteria was less after encapsulation as compared to free bacteria. In addition to this, the free probiotic cells showed poor survival rates under the simulated gastro- intestinal conditions as compared to encapsulated bacteria. This study also proved that sodium alginate was better encapsulating material for probiotics in comparison to carrageenan (93). Another study also investigated the effect of encapsulating bacteria on the sensory properties of yogurt. It was observed that the incorporation of alginate based encapsulated bacteria did not change sensory properties of yogurt including odor as well as color. However, minute textural change was observed in the yogurt containing the encapsulated bacteria. The encapsulated cells resulted in formation of grittiness in the final product and affecting the textural properties of yogurt (112). In addition to this, the incorporation of encapsulated probiotics in yogurt has been observed to increase the viscosity as well as mouth feel properties of the final product. The size of the encapsulated beads containing viable cells is directly linked to sensory characteristics. To illustrate, the microencapsulated beads with larger size are directly linked with more inferior sensory characteristics of the final product. Apart from using single material system for encapsulation, the three layered system for encapsulation has been studied too. To justify, xanthan- chitosan- xanthan multi-layered system was used to encapsulate the probiotic bacteria- *Bifidobacterium* BB01 in yogurt. The viability of encapsulated probiotic bacteria was investigated during the shelf life of yogurt for 21 days. Chitosan was used as the middle layer to encapsulate the target probiotic which was layered between two xanthan layers. The results depicted from this study showed that bacterial viability improved by encapsulation and also helped the bacteria to remain viable during gastric digestion for longer time as compared to free bacteria (94).

There are several probiotics strains which have been studied as microencapsulated bacteria incorporated in yogurt. These probiotics include *L. casei, L. rhamnosus, L. plantarum, B. longum* and *B. bifidum*. Apart from using the encapsulated material for enhancing the survival of probiotics, addition of cereals and milk proteins can increase the survival rates of probiotics too (113). Addition of encapsulated probiotics along with fruit juices often results in increasing the acidic environment of the food matrix and hence affecting the survival rate of probiotics. Hence, the better alternate is to use the nutritionally rich additives to support the viability of encapsulated bacteria. Prebiotics and carbohydrates sources are widely used additive in yogurt which controls the formation of acidic environment during milk fermentation process (114).

### Cheese

Cheese is another dairy product which has been successfully utilized to incorporate the encapsulated probiotic bacteria. The milk proteins present in the cheese matrix provide the suitable environment as well as the nourishment to the probiotic cells. These milk proteins along with other carbohydrate sources are also known to provide the protection to the cells against the harsh stressed conditions of the gut (115). Amongst several variants of cheese, cheddar cheese is considered as the best carrier material for probiotics. It is known that the physiochemical properties of cheddar cheese provide the appropriate environment for survival of bacteria. Low acidic environment of cheddar cheese supports the bacterial survival (116). In one such study, low fat cream cheese has been developed as a successful delivery vehicle for *Lactobacillus rhamnosus*. The free and carbohydrate encapsulated (β-glucan and phytosterol) forms of probiotics were incorporated in the cheese; encapsulation using these sources provided the protection as well as the nourishment. It was observed that addition of encapsulated bacteria showed more survival as compared to free probiotic strains by the end of 35 days. Moreover, the encapsulated bacteria resulted in formation of firm and thicker cheese, with much higher consumer acceptability as compared to the cheese formed by free probiotic bacteria (117). Probiotics are often added as the starter cultures during the cheese making or as additives to enhance the nutritive and sensory properties of the final product. However, addition of probiotics as an additive is always considered as the better option. During the cheese making process, the ripening and storage of cheese often results in lowering the pH of the cheese matrix. As a result of this, the starter culture probiotic bacteria do no survive for long. Encapsulation of probiotics and consuming the bacterial cells as an additive resulted in more viability of probiotic cells (118). Apart from using polysaccharides as the encapsulating materials, milk proteins and wheat starch have been used up to encapsulate the probiotic bacteria. Results revealed that encapsulation of probiotics while using these novel matrices enhanced the probiotic survival under simulated gastro-intestinal conditions. Moreover, the cheese produced using wheat starch and milk protein exhibited acceptable taste, aroma and flavor. It is known that proteins and other carbohydrates sources offer great gelation property, non-reactive nature toward gel as well as offer good buffering capacity; hence favoring the survival of probiotics under harsh environmental conditions (119). Different probiotic strains have been involved in cheese making depending upon the desired flavor and health benefits. There are several probiotics which have been encapsulated using different techniques and have been incorporated into cheese making. The applications of encapsulated probiotics in cheese making have been summarized in Table 3. Another study includes the application of enzyme based gelation using skim milk powder and rennet, skim milk powder and transglutaminase as well as transglutaminase and sodium caseinate, for encapsulating the targeted probiotic strain. For the study, *Lactobacillus paracasei* was used for encapsulation and it was observed that rennet-based encapsulation was most efficient which resulted in more viable cells at the end of storage period and overall better textural properties of the final product (120).

### Bakery

The incorporation of probiotics into bakery products is an emerging area for functional food development domain. The bakery products which have been developed with added probiotics include breads, buns, cookies, soufflés and cakes. However, the main challenge in developing the probiotic rich bakery product includes the maintenance of viability of probiotic cells. Higher temperature during the baking process often results in declining viability of live cells. In addition to this, the longer shelf lives of the bakery products do not always comply with long term viability of probiotic strains. In addition to this, the complexity of food matrix of the bakery product including physio-chemical environment as well as occurrence of milliard reaction upon storage; affects the survival of probiotics (121). Hence, the better alternate is to encapsulate the probiotic strains with suitable materials. In one such study, the use of heat resistant probiotic- *Lactobacillus acidophilus LA-5* was examined. The selected probiotic strain was encapsulated using sodium alginate and additional substances, which enhanced the survival of probiotic bacteria during baking (102). In baking industry, the encapsulated bacteria have been involved in food products using three main techniques: encapsulated bacteria as outer coating, as additive during dough preparation and as additive in creams as well as fillers. To illustrate, *Lactobacillus plantarum ATCC8014* was encapsulated using sodium alginate emulsions which were incorporated during dough baking. The emulsions were further coated with maltodextrin and pectin to provide additional protection to live cells during higher temperatures of baking, leading to successful survival of probiotics under simulated gastric conditions. Moreover, usage of prebiotic components also helped in creating denser network which enhanced the survival of probiotics under harsh conditions (103). Using the first application, the encapsulated probiotics (*Limosilactobacillus fermentum* NKN51 and *Lactobacillus brevis* NKN52) have been applied during the wheat buns making. The encapsulation was achieved by sodium caseinate and chia mucilage. The coating of encapsulated bacteria resulted in increased shelf life of live cells by 3 weeks under cold storage and by 2 weeks at room temperature (104).

### Beverages

The potential health benefits of probiotics have led to its incorporation into the fermented as well as non- fermented beverages. Several fruits and prebiotics rich drinks have been developed with desired probiotic strains. These prebiotic contents include inulin, maltodextrin, lactulose, sorbitol and phytosterol; providing the extra protection to the viable cells. The pH as well as acidity of the fermented and non- fermented beverages affects the probiotic survival. Hence, the better alternate is to encapsulate probiotic bacteria using various techniques like spray drying, emulsions, electro- spraying and extrusion. To illustrate, three probiotic strains namely- *Lactobacillus rhamnosus, Lactobacillus casei* and *Lactobacillus plantarum* have been used to incorporate into the juice mix of acerola and ciriguela. The probiotic bacteria were encapsulated before addition in the fruit juice using spray drying technique. The encapsulating material was a prebiotic rich component- maltodextrin which helped in providing support and nourishment to the probiotic cells during encapsulation. The juice powders produced at the end of the study, showed maximum probiotic viability without affecting the physio- chemical, textural and sensory properties of the final product (107). Another study also demonstrated the incorporation of encapsulating materials like inulin and *Tiliacora triandra* gum to encapsulate *Lactobacillus casei* 01 and *Lactobacillus acidophilus* LA5 for development of probiotic rich maoluang juice. The effects of encapsulated probiotic bacteria have been examined and the results showed that free bacteria were not able to survive for longer duration due to environmental stress of the simulated gastric conditions. However, the encapsulated bacteria showed great survival rates as well as enhanced the accumulation of short chain fatty acids, lactic acid and decline in growth of other pathogenic bacteria too (108). Incorporation of three different prebiotic rich sources has also been used to encapsulate probiotic bacteria using spray drying technique. Encapsulated *L. plantarum* has been used to develop functional litchi juice. The encapsulation using the selected prebiotic strains showed even particle size of the microcapsule, hence enhancing the acceptability of final product. As mentioned earlier, more capsule size results in more grittiness texture of the product. Thus, selection of appropriate particle size as well as considering the viability of probiotics is the most critical step during encapsulation. In addition to this, the selected combination of prebiotics enhanced the survival of encapsulated probiotics under simulated gastric conditions (109).

## Conclusion

Probiotics ingestion is considered a better alternative to medicine and treatments for maintaining a healthier lifestyle. Due to the numerous health-beneficial properties, probiotic bacteria have been incorporated into food items offering consumers a variety of choices from dairy to non-dairy food products. But the major challenge is to maintain the viability of probiotic strains during longer shelf lives of products with desired health beneficial properties exhibited by probiotics upon consumption. Microencapsulation as a technique has a potential to support the survivability of microorganisms in their unique environment. Therefore, if exploited to its full potential it can be used for effective delivery of important health beneficial microbiota via the food matrix. However, despite several studies, the present microencapsulation techniques do not completely support probiotic survival. Proper selection of encapsulating material as well as encapsulation technique is essential criteria to maintain longer viability of probiotic strains. Future studies must include research focused on the exploration of novel cost-effective encapsulating materials and techniques for industrial applications in functional food development. In addition, with increasing number of probiotic strains being explored it is essential that more research be directed to encapsulation of these novel strains as well.

## Author contributions

SSi and SG contributed to conception and design of the study. SSi, RG, SG, SChaw, MS, RT, SU, SSh, and SChan wrote the first draft of the manuscript. SG and PG edited and revised the manuscript. All authors contributed to manuscript revision, read, and approved the submitted version.

## Funding

This work was supported by Jaypee Institute of Information Technology, Noida, for which authors are deeply obliged.

## Conflict of interest

The authors declare that the research was conducted in the absence of any commercial or financial relationships that could be construed as a potential conflict of interest.

## Publisher's note

All claims expressed in this article are solely those of the authors and do not necessarily represent those of their affiliated organizations, or those of the publisher, the editors and the reviewers. Any product that may be evaluated in this article, or claim that may be made by its manufacturer, is not guaranteed or endorsed by the publisher.
