# Supplementary figures and images for: Natural sources and encapsulating materials for probiotics delivery systems: Recent applications and challenges in functional food development
Source: Front Nutr. 2022 Sep 21;9:971784. doi: 10.3389/fnut.2022.971784 (PMC9534265; doi:10.3389/fnut.2022.971784)

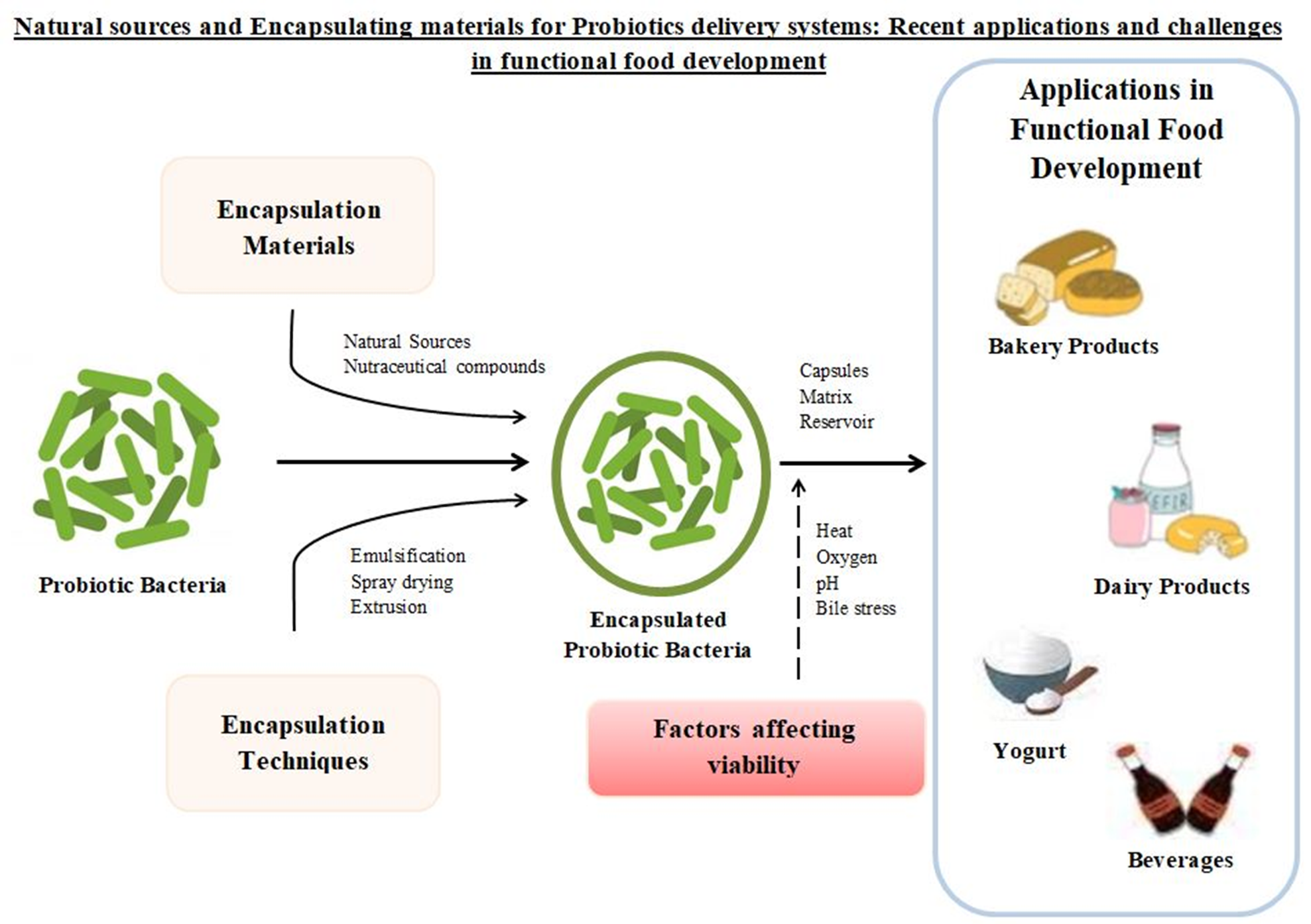

Supplement: Supplementary file 1 [file Image_1.TIFF]
